# Synergistic Enhancement of Antitumor Efficacy by PEGylated Multi-walled Carbon Nanotubes Modified with Cell-Penetrating Peptide TAT

**DOI:** 10.1186/s11671-016-1672-6

**Published:** 2016-10-10

**Authors:** Shanshan Hu, Tong Wang, Xibo Pei, He Cai, Junyu Chen, Xin Zhang, Qianbing Wan, Jian Wang

**Affiliations:** 1 State Key Laboratory of Oral Diseases, West China College of Stomatology, Sichuan University, Chengdu, 610041 China; 2 Department of Prosthodontics, West China College of Stomatology, Sichuan University, Chengdu, 610041 China

**Keywords:** TAT peptide, Multi-walled carbon nanotubes, Doxorubicin, Drug delivery, Antitumor activity

## Abstract

In the present study, a cell-penetrating peptide, the transactivating transcriptional factor (TAT) domain from HIV, was linked to PEGylated multi-walled carbon nanotubes (MWCNTs) to develop a highly effective antitumor drug delivery system. FITC was conjugated on MWCNTs-polyethylene glycol (PEG) and MWCNTs-PEG-TAT to provide fluorescence signal for tracing the cellular uptake of the nanocarrier. After loaded with an anticancer agent, doxorubicin (DOX) via π − π stacking interaction, the physicochemical characteristics, release profile and biological evaluation of the obtained nano-sized drug carrier were investigated. The DOX loaded MWCNTs-PEG and MWCNTs-PEG-TAT drug carriers both displayed appropriate particle size, excellent stability, high drug loading, and pH-dependent drug release profile. Nevertheless, compared with DOX-MWCNTs-PEG, DOX-MWCNTs-PEG-TAT showed improved cell internalization, intracellular distribution and potentiated anticancer efficacy due to the TAT-mediated membrane translocation, endosomal escape and nuclear targeting. Furthermore, the therapeutic efficacy of DOX was not compromised after being conjugated with MWCNTs-PEG-TAT and the proposed nanocarrier was also confirmed to have a good biocompatibility. In conclusion, our results suggested that the unique combination of TAT and MWCNTs as a multifunctional drug delivery system might be a powerful tool for improved anticancer drug development.

## Background

During the last few decades, increasing attention has been paid to multifunctional drug delivery systems using nanotechnology to improve the overall therapeutic efficacy of anticancer activity [1, 2]. Among all those systems, carbon nanotubes (CNTs) are one of the most interesting nanocarries currently under investigation. Ever since their emergence on the nanoplatform, CNTs have shown great promise as novel delivery systems due to their unprecedented advantages, such as ultralight weight, excellent chemical and thermal stability [3], high drug-loading capability, and prolonged circulation time [4]. In particular, CNTs have large surface area and internal cavity that can be functionalized with various kinds of ligands and filled with therapeutic agents to obtain synergistic efficacy of antitumor activity. Doxorubicin (DOX), a DNA interacting drug, has been widely used as the drug of choice in anticancer therapy. However, its clinical utility was hampered by cumulative, dose-limiting cardiotoxicity, myelosuppression, and the developmental drug resistance [5]. As a result, attempts were made to incorporate DOX into CNTs via *π*-*π* stacking to improve therapeutic outcomes [6–8]. Moreover, CNT-based drug delivery systems have also shown great prospect in other various experiments such as delivery of paclitaxel [9] and small interfering RNA [10].

Nevertheless, the application of CNTs in medicine and biology is hampered by their poor solubility in aqueous solutions. Therefore, surface modifications of CNTs including covalent attachment and non-covalent attachment have been used to conquer a lack of solubility and to improve their biocompatibility [11]. Among all the modifications, the covalent link between polyethylene glycol (PEG) and CNTs has lead to stable dispersion and biocompatibility in various biological environments [12].

In order to further improve the intracellular translocation process across the plasma membrane for an enhanced therapeutic effect, a promising approach—the use of cell-penetrating peptides (CPPs) is put forward. CCPs are a family of positively charged short peptides that might be a promising candidate for drug delivery since they were demonstrated to be able to transport attached macromolecules from extracellular space through the cell membrane into cytoplasm effectively in various investigations [13]. The transactivating transcriptional factor (TAT), one of the potential CPPs, is a cationic peptide derived from the human immunodeficiency virus type 1 (HIV-1) [14]. A number of studies have used TAT to transport intracellular cargoes such as liposomes [15], polymers [16], quantum dots [17], micelles [18], and nanoparticles [19]. Moreover, TAT was also found to be capable of taking the DOX-loaded micelles not only into the cells but also to the nucleus [18]. And strategies like PEGylation [20] or charge neutralization [21] have been used to prevent TAT-modified nanocarriers from interacting with non-target tissues and prolong their circulation time in the bloodstream. In addition, TAT has also been shown to exhibit good biocompatibility [22]. However, in spite of the prominence of TAT in the nanotechnology areas, exploration of its application in drug delivery is still at a very early stage. Especially, few papers have addressed the use of CNTs combined with TAT peptide in the field of drug delivery and anticancer therapy.

**Fig. 1:**
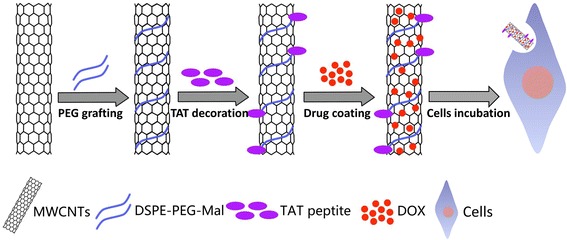
A schematic diagram of the steps involved in preparing DOX-MWCNTs-PEG-TAT for drug delivery

In this study, a novel drug delivery system employing PEGylated multi-walled carbon nanotubes (MWCNTs) was developed with the combination of a cell-penetrating peptide TAT. The synthetic scheme is shown in Fig. 1. Briefly, the raw MWCNTs were first oxidized and the obtained oxidized MWCNTs were then functionalized by 1,2-distearoyl-sn-glycero-3-phosphoethanolamine-N-[methoxy poly(ethylene-glycol)-2000 (DSPE-PEG2000) or 1,2-istearoyl-sn-glycero-3-phosphoethanolamine-N-[poly-(ethylene glycol)-2000]-maleimide (DSPE-PEG2000-Mal). After that, the TAT peptide was conjugated to the maleimide groups of DSPE-PEG2000-Mal by an addition reaction with the sulfhydryl groups of cysteine in TAT. Then, the obtained MWCNTs-PEG and MWCNTs-PEG-TAT were labeled with fluorescein isothiocyanate (FITC) respectively to investigate their intracellular distribution. After being loaded with DOX, the physicochemical characteristics and release profile of the obtained nano-sized drug carrier were investigated. Then, the cellular uptake, intracellular localization, anticancer activity, and apoptosis-inducing capability of the DOX-loaded MWCNTs-PEG and MWCNTs-PEG-TAT were studied in vitro.

## Methods

### Materials

Raw multi-walled carbon nanotubes (MWCNTs) were obtained from Chengdu Organic Chemicals Co. Ltd., with the diameter ranging from 10 to 20 nm and the length ranging from 10 to 30 μm (95 % purity in MWCNTs). TAT (Cys-Tyr-Gly-Arg-Lys-Lys–Arg-Arg-Gln-Arg-Arg-Arg) was purchased from ABbiochem Co. (Shanghai, China). 1,2-Distearoyl-sn-glycero-3-phosphoethanolamine-N-[methoxy poly(ethylene-glycol)-2000] (DSPE-PEG2000) and 1,2-istearoyl-sn-glycero-3-phosphoethanolamine-N-[poly-(ethylene glycol)-2000]-maleimide (DSPE-PEG2000-Mal) were purchased from Laysan Bio Inc. (Arab, Alabama, USA). Doxorubicin (DOX) hydrochloride was obtained from Beijing Huafeng United Technology Co. (Peking, China). Fluorescein isothiocyanate (FITC) was purchased from Sigma-Aldrich (St. Louis, MO, USA). 2-(4-Amidinophenyl)-6-indolecarbamidine dihydrochloride (DAPI) and Annexin V-FITC/PI apoptosis detection kit was obtained from KeyGEN Biotech Co., Ltd. (Shanghai, China). Dulbecco’s modified Eagle’s medium (DMEM), fetal bovine serum (FBS), penicillin-streptomycin solution, and trypsin-EDTA solution were purchased from Hyclone (Logan, Utah, USA). The human osteosarcoma (MG63) cells and human umbilical vein endothelial (HUVEC) cells were supplied by the State Key Laboratory of Oral Diseases (Chengdu, China).

### Preparation and Characterization of MWCNTs

The pristine MWCNTs were initially purified and treated with strong oxidizing acid following the previous method [8]. Briefly, raw MWCNTs were added to a mixture of concentrated nitric acid and sulfuric acid (*V*/*V* = 1:3) for 8 h. After that, the oxidized MWCNTs solution was washed thoroughly with purified water for several times and filtered with polytetrafluoroethylene (PTFE) membrane until the pH value reached neutral. The acquired oxidized MWCNTs were then functionalized by DSPE-PEG2000 or DSPE-PEG2000-Mal according to the previously reported method [23]. Briefly, 10 mg oxidized MWCNTs and DSPE-PEG2000-Mal were dispersed in 10 ml purified water and then sonicated for 2 h at room temperature. The obtained complex (MWCNTs-PEG-Mal) was then purified by Millipore ultrafiltration tube (MWCO = 100 kDa) and dried in vacuum at 60 °C and collected for further use. The MWCNTs-PEG was prepared as MWCNTs-PEG-Mal with the addition of DSPE-PEG2000 instead of DSPE-PEG2000-Mal.

The microstructures and surface morphology of oxidized MWCNTs were characterized by scanning electron microscopy (SEM) (Inspect F50, OR, USA). The fourier transform infrared (FTIR) spectroscopy (Nicolet 6700, WI, USA) was performed to study the functional groups of pristine and oxidized MWCNTs. The Raman spectra (LabRAM HR-800, Longjumeau, France) of nanotubes were recorded with a laser at an excitation wavelength of 632.8 nm. The purity of MWCNTs was investigated by thermogravimetric analysis (TGA) (STA 449/C Jupiter, Germany).

### Conjugation of TAT to DSPE-PEG2000-Mal

The MWCNTs-PEG-Mal was redispersed in PBS and mixed with TAT. After overnight reaction, unbounded excess TAT was removed by Millipore ultrafiltration tube (MWCO = 100 kDa). Then, the resulting complex (MWCNTs-PEG-TAT) was resuspended and stored at 4 °C for further use. MWCNTs, MWCNTs-PEG, MWCNTs-PEG-Mal, and MWCNTs-PEG-TAT were characterized by ^1^H-nuclear magnetic resonance (^1^H-NMR) spectroscopy (Bruker, DRX, USA) at 400 MHz with D_2_O as a solvent.

### FITC Labeling of MWCNTs-PEG-TAT

One milligram per milliliter FITC in dimethyl sulfoxide (DMF) was slowly added to 1 mg/ml MWCNTs-PEG-TAT solution. The mixture was then sonicated in dark for 2 h. Unbounded excess FITC was removed by ultrafiltration (MWCO = 100 kDa) and washed thoroughly with water (over 10 times) until the filtrate becomes color free. The FITC-labeled MWCNTs-PEG-TAT (FITC-MWCNTs-PEG-TAT) was redispersed in PBS (pH 7.4) and stored at 4 °C in the dark. The FITC-labeled MWCNTs-PEG (FITC-MWCNTs-PEG) was prepared as above with MWCNTs-PEG instead of MWCNTs-PEG-TAT. Then, the percentages of DSPE-PEG2000, DSPE-PEG2000-Mal, TAT, and FITC linked to MWCNTs were quantified by TGA (STA 449/C Jupiter, Germany) with a heating rate of 10 °C/min from 30 to 800 °C, respectively.

### DOX Loaded onto MWCNTs-PEG-TAT

DOX loaded onto MWCNTs-PEG-TAT was performed as follows: 10 mg MWCNTs-PEG-TAT was dispersed in PBS (pH 9.0) [6, 7]. Then, 10 mg DOX was slowly added into the MWCNTs-PEG-TAT solution and sonicated for 2 h in a dark environment. Unbounded excess DOX was removed by ultrafiltration tube (MWCO = 100 kDa) and washed thoroughly with purified water (over 10 times) until the filtrate becomes color free. The DOX-loaded MWCNT-PEG-TAT (DOX-MWCNTs-PEG-TAT) was resuspended in PBS (pH 7.4) and stored at 4 °C in darkness. The unbounded DOX was measured at an absorbance of 490 nm by microplate reader (MK3, Thermo, USA). The calibration curve was also recorded under the same conditions, allowing the amount of unbounded DOX to be calculated. The DOX-loaded MWCNTs-PEG (DOX-MWCNTs-PEG) was prepared as above with MWCNTs-PEG instead of MWCNTs-PEG-TAT. Then, the UV-Vis absorption spectra of MWCNTs-PEG, MWCNTs-PEG-TAT, free DOX, DOX-MWCNTs-PEG, and DOX-MWCNTs-PEG-TAT were measured to verify the loading of DOX onto MWCNTs by a UV-Vis spectrophotometer (UV-3600, SHIMADZU, Kyoto, Japan). The DOX loading efficiency (DL%) was calculated using the following formula:

$$ \mathrm{D}\mathrm{L}\%=\frac{\mathrm{Weight}\kern0.5em \mathrm{of}\kern0.5em \mathrm{loaded}\kern0.5em \mathrm{D}\mathrm{O}\mathrm{X}\hbox{-} \mathrm{Weight}\kern0.5em \mathrm{of}\kern0.5em \mathrm{free}\kern0.5em \mathrm{D}\mathrm{O}\mathrm{X}}{\mathrm{Weight}\kern0.5em \mathrm{of}\kern0.5em \mathrm{loaded}\kern0.5em \mathrm{D}\mathrm{O}\mathrm{X}}\times 100\% $$

### Particle Size and Zeta Potential

The mean particle size and zeta potential of MWCNTs-PEG, MWCNTs-PEG-TAT, DOX-MWCNTs-PEG, and DOX-MWCNTs-PEG-TAT were measured by a Malvern Zetasizer APS ZEN3600 instrument (Malvern Instruments Ltd., UK) at 25 °C.

### In Vitro Drug Release of DOX

DOX release behaviors from MWCNT complexes were investigated according to the previously reported method [7]. Briefly, DOX-MWCNTs-PEG and DOX-MWCNTs-PEG-TAT conjugates were studied in PBS (pH 5.3 and pH 7.4) as release medium. The MWCNT conjugates were filled in dialysis membrane (MWCO = 1 k Da) and then immersed in 200 ml of PBS with stirring at 100 rpm at 37 °C in the dark. At definite time points, 2 ml of the medium was withdrawn and equivalent volumes of PBS were replaced. The concentration of released DOX was estimated by microplate reader.

### Intracellular Tracking of FITC-MWCNTs-PEG-TAT

MG63 and HUVEC cells were plated in 6-well plates, with the cells being seeded for 24 h before incubation. Then, the cells were incubated in DMEM for 30 min and treated with FITC-MWCNTs-PEG or FITC-MWCNTs-PEG-TAT at MWCNT concentration of 20 μg/ml for 2 h. After that, cells were washed with PBS three times followed by soaking for 15 min in 4 % paraformaldehyde and washed with PBS. The nuclei were stained with DAPI according to the operation manuals. The stained cells were immediately observed using a fluorescence microscope (TE 2000-U, Nikon, Japan).

### Intracellular Distribution of DOX-MWCNTs-PEG-TAT

MG63 and HUVEC cells were first cultured overnight to allow attachment. Then, cells were incubated with free DOX, DOX-MWCNTs-PEG and DOX-MWCNTs-PEG-TAT at DOX concentration of 10 μg/ml for 2 h at 37 °C in DMEM respectively. After incubation, the cells were washed repeatedly with PBS three times followed by soaking for 15 min in 4 % paraformaldehyde and washed with PBS three times. The nuclei were stained with DAPI according to the operation manuals. The stained cells were then observed by a fluorescence microscope.

### In Vitro Cytotoxicity Assay

MG63 cells and HUVEC cells were used to evaluate the cytotoxicity of MWCNTs-PEG and MWCNTs-PEG-TAT using the CCK-8 cell viability assay. Briefly, cells were seeded in 96-well plates with a density of 20,000 cells/well and allowed to adhere for 24 h prior to assay. Then, the cells were treated with MWCNTs-PEG or MWCNTs-PEG-TAT in culture medium for another 24 h. Subsequently, the culture medium was removed and replaced by 100 μl of fresh medium and 10 μl of CCK-8 solution and incubated for 2 h. Then, the OD value at 450 nm was detected using a microplate reader. Furthermore, the cell morphology of MG63 and HUVEC cells treated with MWCNTs-PEG or MWCNTs-PEG-TAT for 24 h at MWCNT concentration of 50 μg/ml was observed by light microscopy (Olympus IX, Japan).

The cytotoxicities of free DOX, DOX-MWCNTs-PEG, and DOX-MWCNTs-PEG-TAT were also investigated by the CCK-8 cell viability assay as above using MG63 cells.

### Apoptosis Study

To study the apoptosis-inducing capability of DOX-MWCNTs-PEG-TAT, MG63 cells were incubated with 10 μg/ml DOX-MWCNTs-PEG-TAT for 2, 8, 24, and 72 h at 37 °C in DMEM, respectively. After incubation, the cells were washed repeatedly with sterilized PBS and analyzed by fluorescence microscopy.

Quantitative analysis was performed using Annexin V-FITC/PI double staining. Briefly, MG63 cells were seeded at a density of 5 × 10^5^ cells in 6-well plates and incubated overnight before treatment. Then, the MG63 cells were treated with 10 μg/ml DOX-MWCNTs-PEG-TAT for 2, 8, 24, and 72 h, respectively. After that, the cells were trypsinized, washed with cold PBS, suspended in 400 μl of binding buffer, and stained with 5 μl Annexin V-FITC for 15 min and 5 μl propidium iodide (PI) for 5 min at 4 °C in the dark. At the end of the treatment, the cells were analyzed by a flow cytometer (Beckman Coulter, Fullerton, CA, USA).

### Statistical Analysis

The obtained results were expressed as mean ± standard deviation (SD) of triplicate. The statistical analysis was analyzed using one-way ANOVA. A probability of *P* < 0.05 was considered statistically significant.

## Results

### Characterization of MWCNTs

**Fig. 2:**
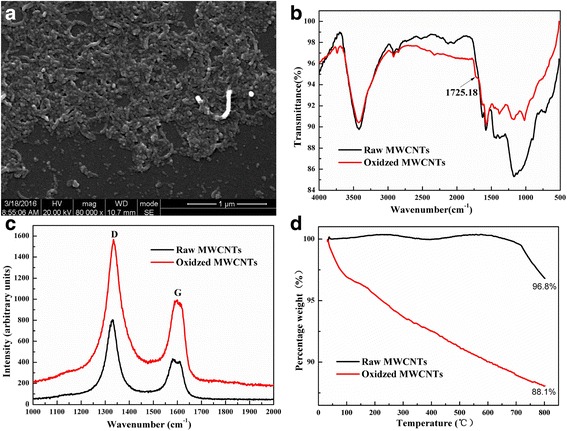
**a** The SEM image of oxidized MWCNTs. **b** The FTIR spectra of raw MWCNTs and oxidized MWCNTs. **c** The Raman spectra of raw MWCNTs and oxidized MWCNTs. **d** The TGA results of raw MWCNTs and oxidized MWCNTs in nitrogen atmosphere

The morphology of the oxidized MWCNTs was characterized by SEM (Fig. 2a). The results showed that the length of oxidized MWCNTs decreased apparently compared with the raw MWCNTs (10 to 30 μm). The FTIR spectra of raw MWCNTs and oxidized MWCNTs were shown in Fig. 2b. The peaks at 1575.24 and 3423.62 cm^−1^ shown in both spectra were ascribed to the presence of carbon residue on the CNT surface and O–H stretching vibration, respectively. The characteristic peak at 1725.18 cm^−1^ indicated the introduction of carboxylic groups of oxidized MWCNTs, which was extraordinarily weak in raw MWCNTs. The Raman spectra of raw and oxidized MWCNTs were shown in Fig. 2c. As indicated in the graph, raw MWCNTs showed the Raman shift at 1329.72 and at 1579.76 cm^–1^, which corresponds to the D band (disorder-induced bands) and G band (graphite-like modes), respectively [24]. However, the D band was shifted to 1334.49 and G band to 1598.37 cm^–1^ after the carboxylation of MWCNTs. To quantify the concentration of carboxylic groups present on the surface of CNTs, thermogravimetric analysis (TGA) of was also conducted under nitrogen atmosphere. It is clear that the weight loss of oxidized MWCNTs is only about 3.2 % at the inflection point (800 °C), while the oxidized MWCNTs has a weight loss of 11.9 % (Fig. 2d).

### Synthesis and Characterization of MWCNT Composites

**Fig. 3:**
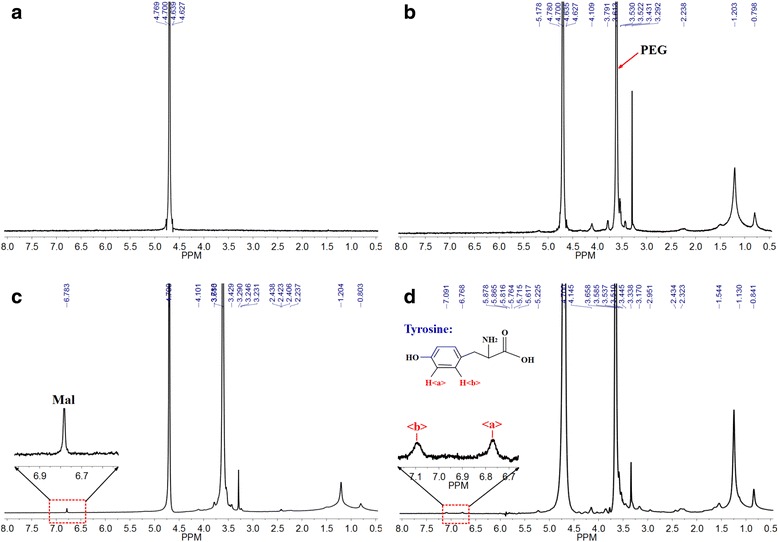
The ^1^H NMR spectra of **a** MWCNTs, **b** MWCNTs-PEG, **c** MWCNTs-PEG-Mal, and **d** MWCNTs-PEG-TAT

The scheme of MWCNTs-PEG-TAT was shown in Fig. 1, and the structure of the resulting complex was verified by ^1^H NMR. As illustrated in Fig. 3a, the characteristic resonance peak of 4.700 ppm was the solvent residual peak. The characteristic peaks of PEG (3.613 ppm) and Mal (6.783 ppm) shown in Fig. 3b,c, respectively, indicated the successful synthesis of MWCNTs-PEG and MWCNTs-PEG-Mal. After conjugation of TAT peptide to MWCNTs-PEG-Mal, two characteristic peaks at 6.768 ppm (signal a) and 7.091 ppm (signal b) were observed in Fig. 3d.

**Fig. 4:**
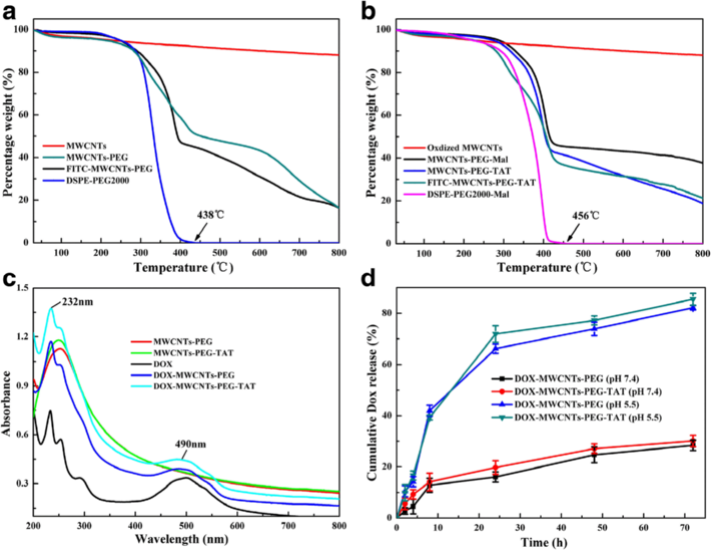
**a** The TGA curves of oxidized MWCNTs, MWCNTs-PEG, FITC-MWCNTs-PEG, and DSPE-PEG2000, respectively. **b** The TGA curves of oxidized MWCNTs, MWCNTs-PEG-Mal, MWCNTs-PEG-TAT, FITC-MWCNTs-PEG-TAT, and DSPE-PEG2000-Mal, respectively. **c** UV-Vis absorption spectra of DOX and different MWCNTs complexes. **d** Drug release of DOX loaded MWCNTs-PEG and MWCNTs-PEG-TAT under different pH

TGA was used to quantitatively analyze the percentages of DSPE-PEG2000, DSPE-PEG2000-Mal, TAT, and FITC linked onto the surface of MWCNTs, respectively. As presented from Fig. 4a, DSPE-PEG2000 has 100 % weight loss at the temperature of 438 °C, which could be selected as the inflection point to calculate the weight losses of MWCNTs after different modifications [25]. It could be seen that at this inflection point (438 °C), oxidized MWCNTs, MWCNTs-PEG, and FITC-MWCNTs-PEG had weight losses of 8, 49, and 55 %, respectively. Therefore, by subtracting the weight loss of unmodified oxidized MWCNTs and MWCNTs-PEG, the conjugating percentages of DSPE-PEG2000 and FITC were estimated to be 41 and 6 %, respectively. Similarly, in the case of FITC-MWCNTs-PEG-TAT composite (Fig. 4b), as oxidized MWCNTs, MWCNTs-PEG-Mal, MWCNTs-PEG-TAT, and FITC-MWCNTs-PEG-TAT had weight losses of 8, 55, 59, and 64 %, respectively, at the inflection point (456 °C), the grafting percentages of DSPE-PEG2000-Mal, TAT, and FITC could be estimated to be 47, 4, and 5 %, respectively.

### DOX Loading onto MWCNTs-PEG-TAT

DOX loading could be monitored by UV-Vis absorption spectroscopy because DOX in water displays two main characteristic absorptions at around 490 and 232 nm. As shown in Fig. 4c, like free DOX, DOX-MWCNTs-PEG, and DOX-MWCNTs-PEG-TAT also showed that these two absorption peaks after conjugated with DOX when the pH is 9.0, while MWCNTs-PEG and MWCNTs-PEG-TAT appeared no peaks in the corresponding areas. Moreover, DOX-MWCNTs-PEG and DOX-MWCNTs-PEG-TAT prepared in this study exhibited drug-loading efficiency of 97.3 and 98.2 %, respectively (Table 1).

**Table 1:** Physiochemical characterization of different MWCNTs complexes

Group	Particle size (nm)	Polydispersity index	Zeta potential (mV)	DOX loading efficiency (%)
MWCNTs-PEG	89.39	0.343	−8.46	–
MWCNTs-PEG-TAT	96.26	0.394	−9.03	–
DOX-MWCNTs-PEG	135.24	0.290	−3.53	97.3
DOX-MWCNTs-PEG-TAT	145.24	0.339	−4.96	98.2

In this study, the average particle size of DOX-MWCNTs-PEG and DOX-MWCNTs-PEG-TAT were found to be 135.24 and 145.24 nm, respectively (Table 1). Moreover, the two complexes had low polydispersity indexes (PDIs) of 0.290 and 0.339, respectively (Table 1), indicating homogeneity in their dispersion state. The zeta potentials of MWCNTs-PEG, MWCNTs-PEG-TAT, DOX-MWCNTs-PEG, and DOX-MWCNTs-PEG-TAT were found to be −8.46, −9.03, −3.53, and −4.96 mV, respectively (Table 1).

### In Vitro Drug Release of DOX

The results of in vitro DOX release from DOX-MWCNTs-PEG and DOX-MWCNTs-PEG-TAT at 37 °C in PBS at pH 7.4 and 5.5 were presented in Fig. 4d. The drug release curves showed that the two complexes displayed similar accumulative release curves. A slow and controlled release manner could be seen at pH 7.4, to the extent of nearly 30 % in 72 h, while a significant faster release was observed in pH 5.5, with the accumulative release achieved high percentage of above 80 % within the same period.

### Intracellular Tracking of FITC-Labeled MWCNTs-PEG-TAT

**Fig. 5:**
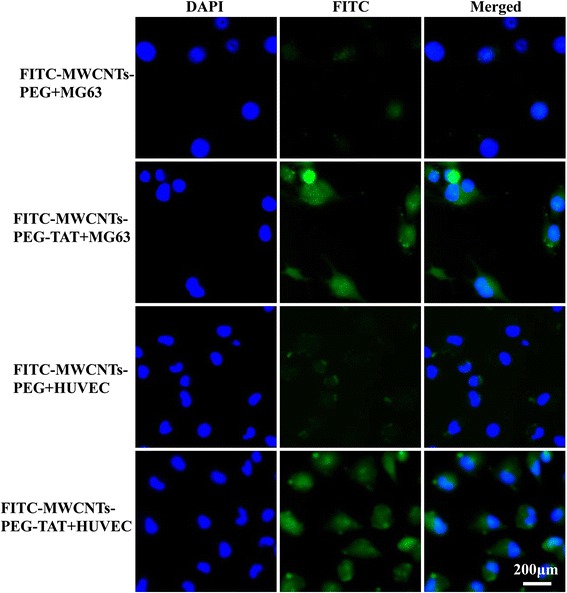
Intracellular fluorescence distribution in MG63 and HUVEC cells after incubated with FITC-MWCNTs-PEG or FITC-MWCNTs-PEG-TAT at MWCNTs concentration of 20 μg/ml for 2 h.

Uptake of FITC-MWCNTs-PEG and FITC-MWCNTs-PEG-TAT by MG63 and HUVEC cells were examined. Fluorescence microscopy was applied to track the intracellular distribution of the FITC-labeled MWCNTs-PEG and MWCNTs-PEG-TAT inside cells. Figure 5 showed the fluorescent distribution of FITC-MWCNTs-PEG and FITC-MWCNTs-PEG-TAT in MG63 and HUVEC cells, respectively. The green fluorescence indicated the distribution of FITC-MWCNTs-PEG or FITC-MWCNTs-PEG-TAT, and the blue fluorescence indicated the nuclei stained by DAPI. Both in MG63 and HUVEC cells, the FITC fluorescence was significantly higher in cells incubated with FITC-MWCNTs-PEG-TAT than that of FITC-MWCNTs-PEG.

### Cellular Uptake and Intracellular Distribution of DOX-MWCNTs-PEG-TAT

**Fig. 6:**
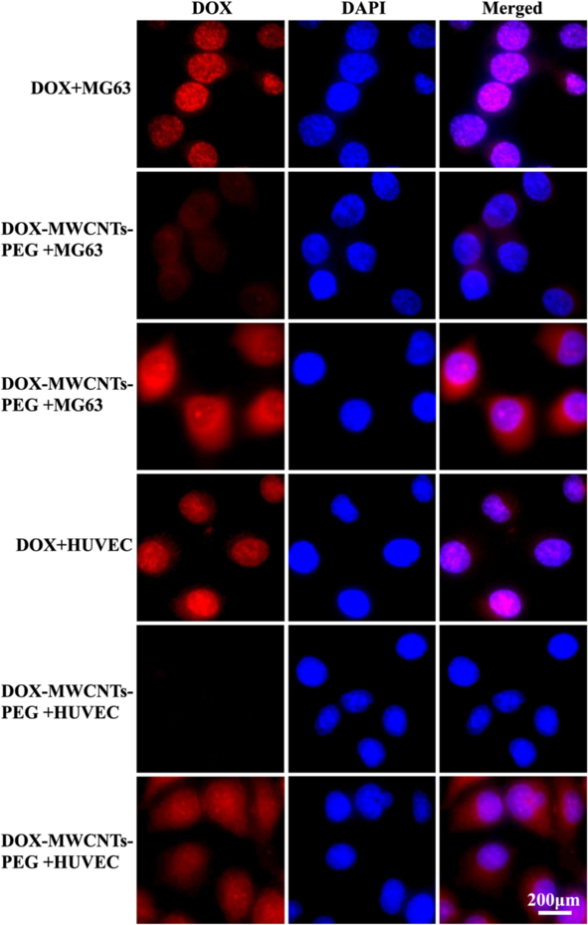
Fluorescence images of MG63 and HUVEC cells incubated with free DOX, DOX-MWCNTs-PEG, and DOX-MWCNTs-PEG-TAT for 2 h at DOX concentration of 10 μg/ml

In order to further investigate the role of TAT peptide and the intracellular and intranuclear behavior of MWCNTs-PEG-TAT after drug loading, the free DOX, DOX-MWCNTs-PEG, and DOX-MWCNTs-PEG-TAT were incubated with MG63 and HUVEC cells and analyzed by fluorescence microscopy since the red fluorescence of the DOX and the blue fluorescence of the DAPI enabled the visualization of the cellular uptake and intracellular distribution of the DOX and DOX-loaded MWCNT complexes. As shown in Fig. 6, DOX accumulated in the perinuclear region and cell nuclei of MG63 and HUVEC cells following 2-h incubation with free DOX. However, the red fluorescence of DOX in the DOX-MWCNTs-PEG and DOX-MWCNTs-PEG-TAT complexes spread all over the cells, primarily in the cytoplasm with a small amount in the nuclei.

Moreover, in contrast to the very slight red fluorescence in DOX-MWCNTs-PEG, DOX-MWCNTs-PEG-TAT exhibited much stronger red fluorescence signals inside MG63 and HUVEC cells and displayed a more diffuse distribution, indicating that the TAT conjugated nanotubes were taken up more efficiently into MG63 and HUVEC cells.

### In Vitro Cytotoxicity Assay

**Fig. 7:**
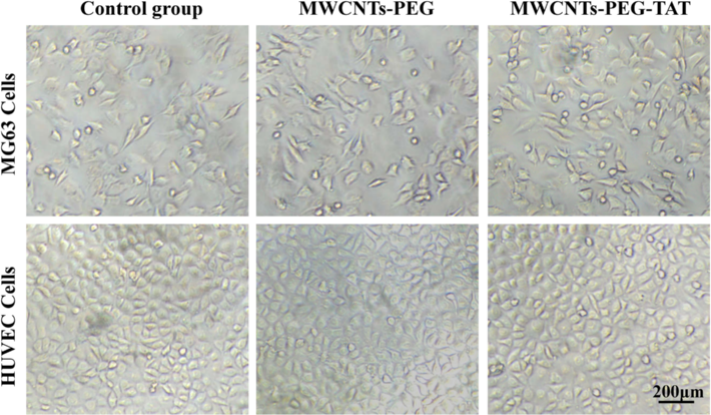
Light microscopy images of MG63 and HUVEC cells treated with MWCNTs-PEG or MWCNTs-PEG-TAT for 24 h at MWCNTs concentration of 50 μg/ml

**Fig. 8:**
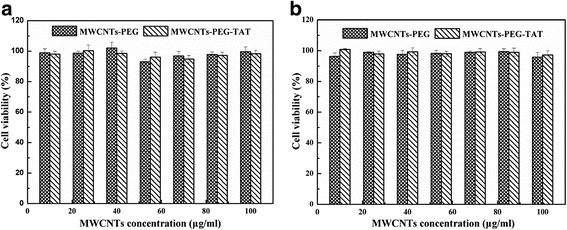
Viability of **a** MG63 cells and **b** HUVEC cells treated with MWCNTs-PEG and MWCNTs-PEG-TAT at different concentrations for 24 h

As shown in Fig. 7, MG63 and HUVEC cells continuously incubated with MWCNTs-PEG and MWCNTs-PEG-TAT for 24 h showed no detectable differences in cell morphology. Moreover, the quantitative studies examining the viability of these treated cells by the CCK-8 assay also showed that the cell viability all remained above 90 % at different concentrations, and there were no significant differences between MWCNTs-PEG and MWCNTs-PEG-TAT at any of the given concentrations (Fig. 8). Therefore, these results proved good biocompatibility and biological safety of MWCNTs-PEG and MWCNTs-PEG-TAT.

**Fig. 9:**
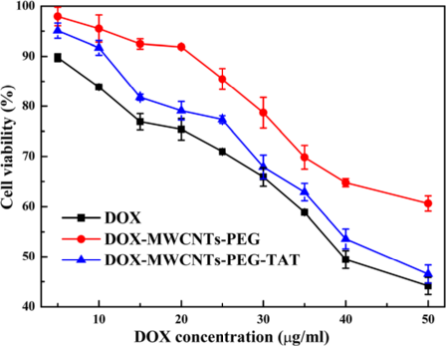
Viability of MG63 cells treated with free DOX, DOX-MWCNTs-PEG, and DOX-MWCNTs-PEG-TAT for 24 h

After being loaded with DOX, the cytotoxicity assay towards MG63 cells was also conducted to evaluate the antitumor activity of the drug-loaded MWCNTs and the results were shown in Fig. 9. The IC50 (the concentration of drug resulting in 50 % cell killing) values of free DOX, DOX-MWCNTs-PEG, and DOX-MWCNTs-PEG-TAT were 43.38 ± 1.08, 59.81 ± 1.15, and 46.20 ± 2.33 μg/ml, respectively.

### Apoptosis Study

**Fig. 10:**
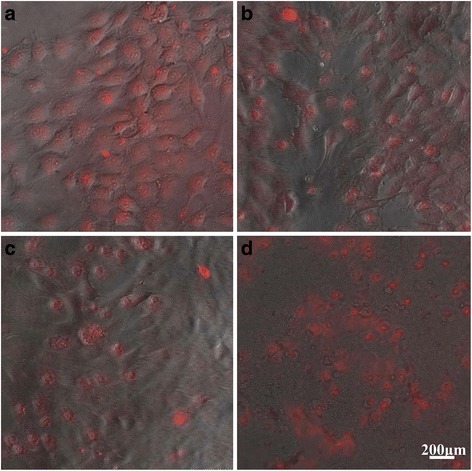
Fluorescence images of MG63 cells treated with DOX-MWCNTs-PEG-TAT at DOX concentration of 10 μg/ml for **a** 2 h, **b** 8 h, **c** 24 h, and **d** 72 h

To study the apoptosis-inducing capability of DOX-MWCNTs-PEG-TAT, the MG63 cell morphology change and intracellular localization of DOX after treatment for 2, 8, 24, and 72 h were recorded respectively by the fluorescence images. As shown in Fig. 10, DOX-MWCNTs-PEG-TAT conjugates were efficiently taken up by MG63 cells and rapidly localized in the cytoplasm and nucleus within 2 h (Fig. 10a) with no detectable differences in cell morphology. However, after 8 h (Fig. 10b), more DOX accumulated in the perinuclear region and cell nuclei and induced appreciable cell morphology changes, which was indicative of the fact that the cells had undergone apoptosis. After 24-h (Fig. 10c) incubation with DOX-MWCNTs-PEG-TAT, nearly all DOX was observed in the nuclei and the cancer cells underwent extensive apoptosis, while after 72 h (Fig. 10d), nearly all cells died and no cell morphology could be observed.

**Fig. 11:**
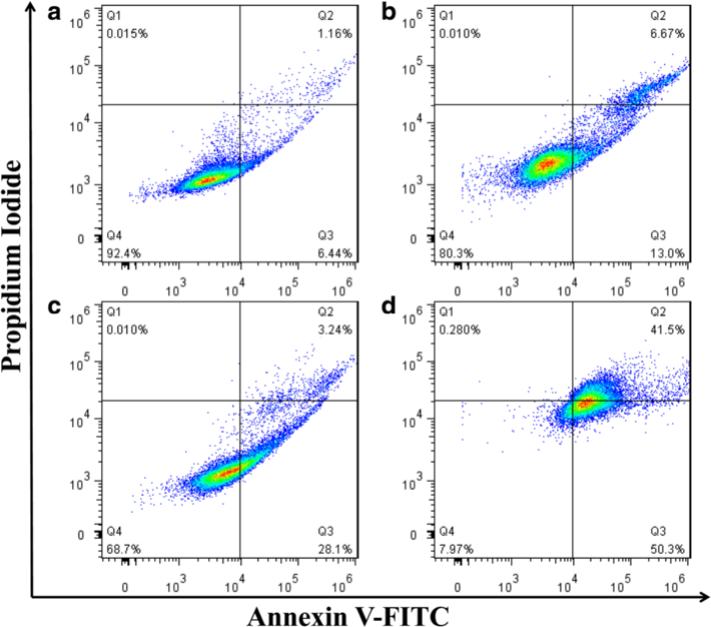
Flow cytometric analysis of cell apoptosis using Annexin V-FITC and PI staining. The MG63 cells were treated with DOX-MWCNTs-PEG-TAT at DOX concentration of 10 μg/ml for **a** 2 h, **b** 8 h, **c** 24 h, and **d** 72 h

In order to further study the cell apoptosis-inducing capability of DOX-MWCNTs-PEG-TAT, quantitative analysis was performed by staining with Annexin V FITC and PI in MG63 cancer cells. The scatter plot has four quadrants: viable cells (Annexin V− PI−, Q4), early apoptotic cells (Annexin V+ PI−, Q3), late apoptotic cells (Annexin V+ PI+, Q2), and necrotic cells (Annexin V− PI+, Q1). As seen in Fig. 11, after incubation with 10 μg/ml DOX-MWCNTs-PEG-TAT for 2 h, only a small number of apoptotic cells were observed. While after 8 h, approximately 13.0 % of cells were in early apoptosis stage and 6.67 % of cells were in late apoptosis stage. After 24 h of incubation, up to 31.34 % cancer cells underwent either early apoptosis or late apoptosis. As expected, after 72 h, most MG63 cells underwent apoptosis or necrosis, with 92.08 % of cells observed in the Annexin V positive or PI positive quadrants (Q1 + Q2 + Q3).

## Discussion

The surface of CNTs plays a vital role in the research and development of drug delivery system. As raw nanotubes disperse poorly in aqueous solutions and often are too long to be able to enter most cells and may be toxic, techniques such as oxidation are employed to introduce the hydrophilic functional groups at the side wall of the CNTs via strong oxidizing acid method [8]. In this study, the carboxylic groups were generated onto the MWCNTs surface by the mixed acid reflux and the oxidized MWCNTs were verified by FTIR, Raman spectra, and TGA. The characteristic peak at 1725.18 cm^−1^ in the FTIR spectrum indicated the introduction of carboxylic groups of oxidized MWCNTs (Fig. 2b). Moreover, Raman spectroscopy was not only able to give the information about the hybridization state and defect concentration of the nanotubes but also could offer information about the slight structural changes of the nanotubes [26]. The weight loss of oxidized MWCNTs shown in the TGA spectrum was partly because water is absorbed from the air, demonstrating the highly hygroscopic nature of the oxidized nanotubes, and partly attributed to the consumption of carboxylic groups [27, 28]. Altogether, these results demonstrated the presence of carboxylic acid groups at the surface of the oxidized MWCNTs, which would not only conquer the poor solubility and improve the biocompatibility of oxidized MWCNTs but also contribute to the subsequent PEG grafting.

The ^1^H NMR results confirmed the successful synthesis of MWCNTs-PEG-TAT. As the maleimide groups could selectively undergo an addition reaction with sulfhydryl groups [29], the DSPE-PEG-Mal conjugate would be able to react with cysteine in TAT and form covalent bonding. Moreover, due to the influence of hydroxyl on the benzene ring in tyrosine of TAT peptide, the proton in the ortho-position and meso-position of the benzene ring would be different, which would result in two peaks around 7.27 ppm (the standard chemical shift of benzene ring) in the ^1^H NMR spectrum. Therefore, in this study, the peaks at 6.768 ppm (signal a) and 7.091 ppm (signal b) in Fig. 3d signified the successful conjugation of TAT peptide onto MWCNTs-PEG-Mal [30].

As a model anticancer drug, DOX has been shown to be able to be adsorbed onto the sidewalls of CNTs due to their high surface area and conjugation structure via *π*-*π* stacking [6]. Furthermore, the hydrophilicity behavior of DOX is pH-dependent, which would be beneficial for the *π*-*π* stacking between DOX and MWCNTs. Consequently, the UV-Vis absorption spectroscopy results indicated the successful loading of DOX on MWCNTs-PEG and MWCNTs-PEG-TAT, and the high drug-loading efficiency of the nanocarriers was due to the aromatic structure and ultrahigh surface areas of MWCNTs.

The physicochemical characteristics are crucial factors to the therapeutic performance of nanocarriers in drug delivery systems. The particle size would be a prerequisite since it influences drug efficacy and pharmacokinetics [31]. The size between 100 and 200 nm was demonstrated to be the best size for enhanced permeability and retention (EPR) effect [32]. In this study, the average particle size of DOX-MWCNTs-PEG and DOX-MWCNTs-PEG-TAT were both perfectly controlled between 100 and 200 nm. Moreover, as electric charges repel each other, potentials on surface of the nanoparticles can prevent them from flocculation and keep them stable. Therefore, zeta potential was measured to verify the stability of the nanocarries. In our case, the modified MWCNTs were all slightly negatively charged (Table 1), which would be beneficial to their physical stability. As a result, the appropriate particle size and excellent stability suggested that DOX-MWCNTs-PEG and DOX-MWCNTs-PEG-TAT would be suitable for drug delivery.

For drug delivery applications, the conjugated DOX on nanocarries should be able to be released to exert its therapeutic efficacy. As expected, the release profile of DOX from MWCNTs complexes should also be pH-dependent. This pH-triggered DOX release was attributed to the increased hydrophilicity and higher solubility of DOX at lower pH, which would be helpful to overcome the *π*-*π* stacking between the DOX and MWCNTs complexes and thus facilitates the detachment of DOX from the nanotubes [30]. As a result, this particular release profile would be beneficial for drug delivery applications, since DOX would be released more in acidic tumor sites than in the normal tissues, which could increase intercellular distribution of DOX in tumor cells and benefit the anticancer efficacy of MWCNTs nanocarriers [33].

The intracellular tracking of free DOX, DOX-MWCNTs-PEG, and DOX-MWCNTs-PEG-TAT in MG63 and HUVEC cells reflected the disparate mechanisms by which they gained cellular entry (Fig. 6). Free DOX could easily enter into cells and access the cell nucleus via passive diffusion [34], while DOX-MWCNTs-PEG and DOX-MWCNTs-PEG-TAT relied on an active transport mechanism for cellular entry and then released the drug in the cytoplasm and diffused into nuclei [35]. Moreover, the obviously higher red fluorescence distribution in DOX-MWCNTs-PEG-TAT than that of DOX-MWCNTs-PEG clearly indicated the improved efficacy of the former. There may be many reasons lying behind this phenomenon. (1) The TAT peptide enhanced the translocation across the cellular membrane. (2) The TAT-mediated cytoplasmic uptake of drug conjugates can deliver the cargo directly at periphery of the nucleus and undergo endosomal escape [36], which would increase the cytosolic drug concentration. (3) The TAT peptide was reported to possess DNA-binding abilities [37]. Therefore, the improved nuclear translocation evidenced by the high nuclear accumulation of DOX-MWCNTs-PEG-TAT in the MG63 and HUVEC cells suggested that TAT may serve as a synergistic factor for DNA targeting.

When compared with free DOX, the DOX-loaded MWCNTs-PEG and MWCNTs-PEG-TAT both showed a lower cytotoxicity at different concentrations (Fig. 9), which was consistent with other studies of DOX bound to nanocarries [26, 38]. This phenomenon could be explained by the reason that free DOX was a hydrophilic anticancer drug and could be quickly taken up by passive diffusion and translocate to the nucleus to act as a DNA intercalator and inhibitor of topoisomerase II [39]. However, drug molecules conjugated on MWCNTs released only after encountering lower pH inside cells and so smaller amounts of drug could reach the nucleus to exert a cytotoxic effect. Moreover, the cell vitality further decreased with increasing of concentration, suggesting that sufficient DOX was released from the DOX-loaded nanotubes and was translocated into nuclei during the incubation time.

However, unlike the DOX-MWCNTs-PEG which did not result in appreciable cytotoxicity at the administered concentrations, DOX-MWCNTs-PEG-TAT exhibited much higher cytotoxicity and was even comparable to free DOX especially at relatively high doses, indicating that the antiproliferative effect of the drug-loaded MWCNTs was significantly increased by the modification of TAT. It was probably because that upon endocytosis, low endosomal pH could trigger the TAT insertion into the endosomal membrane and facilitate its translocation to the cytosol, which could increase the cytosolic concentration of TAT modified drug nanocarriers [36]. Furthermore, the nuclear translocation capability of TAT peptide [40] might also contribute to the improved anticancer efficacy of DOX-MWCNTs-PEG-TAT since DOX is a DNA interacting drug and causes cell apoptosis by damaging the DNA structure [41].

Moreover, based on the in vitro cytotoxicity and apoptosis assays, it could be demonstrated that the DOX-MWCNTs-PEG-TAT could result in obvious antiproliferative and apoptosis effects to cancer cells. Therefore, it was believed that the conjugation of TAT on MWCNTs composites may potentially improve the therapeutic efficacy in anticancer research.

Consequently, these results indicated that, like free DOX, the DOX-MWCNTs-PEG-TAT could efficiently kill cancer cells via the induction of cell apoptosis. Furthermore, the therapeutic efficacy of DOX was not compromised after being conjugated with MWCNTs-PEG-TAT, which means that DOX can be released efficiently from the internalized nanocarriers in the cytoplasmic region and enters the nucleus to induce cell death when encountered in intracellular endosome of low pH.

## Conclusions

In conclusion, a highly effective drug delivery system based on cell-penetrating peptide TAT modified with PEGylated MWCNTs was developed, characterized, and applied to augment the antitumor activity of DOX to cancer cells. The results showed that the TAT-modified MWCNTs displayed appropriate characteristics of nano-sized drug carriers and showed potentiated anticancer efficacy than non-TAT-modified MWCNTs. However, TAT peptide is a non-specific ligand, which could penetrate any cell and limits its applications especially in targeted drug delivery. Therefore, improvements could be made by grafting additional targeting ligand for their targeting to defined cell compartments. Despite this, the results in the present paper suggested that the unique combination of TAT and MWCNTs as a multifunctional drug delivery system is potent in regressing tumor growth and might be a powerful tool for improved anticancer drug development.

## Abbreviations

^1^H-NMR: ^1^H-nuclear magnetic resonance
CPPs: Cell-penetrating peptides
DAPI: 2-(4-Amidinophenyl)-6-indolecarbamidine dihydrochloride
DMEM: Dulbecco’s modified Eagle medium
DMF: Dimethyl sulfoxide
DOX: Doxorubicin
DSPE-PEG2000: 1,2-distearoyl-sn-glycero-3-phosphoethanolamine-N-[methoxy poly(ethylene-glycol)-2000
DSPE-PEG2000-Mal: 1,2-istearoyl-sn-glycero-3-phosphoethanolamine-N-[poly-(ethylene glycol)-2000]-maleimide
FBS: Fetal bovine serum
FITC: Fluorescein isothiocyanate
FTIR: Fourier transform infrared
HIV-1: Human immunodeficiency virus type 1
MWCNTs: Multi-walled carbon nanotubes
PDI: Polydispersity index
PI: Propidium iodide
PTFE: Polytetrafluoroethylene membrane
SEM: Scanning electron microscopy
TAT: Transactivating transcriptional factor
TGA: Thermogravimetric analysis
